# Concerns Around Opposition to the Green Pass in Italy: Social Listening Analysis by Using a Mixed Methods Approach

**DOI:** 10.2196/34385

**Published:** 2022-02-16

**Authors:** Giovanni Spitale, Nikola Biller-Andorno, Federico Germani

**Affiliations:** 1 Institute of Biomedical Ethics and History of Medicine University of Zurich Zurich Switzerland

**Keywords:** green pass, COVID-19, COVID-19 pandemic, vaccines, vaccination hesitancy, freedom, social listening, social media, infodemic, bioethics, telegram

## Abstract

**Background:**

The recent introduction of COVID-19 certificates in several countries, including the introduction of the European green pass, has been met with protests and concerns by a fraction of the population. In Italy, the green pass has been used as a nudging measure to incentivize vaccinations because a valid green pass is needed to enter restaurants, bars, museums, or stadiums. As of December 2021, a valid green pass can be obtained by being fully vaccinated with an approved vaccine, recovered from COVID-19, or tested. However, a green pass obtained with a test has a short validity (48 hours for the rapid test, 72 hours for the polymerase chain reaction test) and does not allow access to several indoor public places.

**Objective:**

This study aims to understand and describe the concerns of individuals opposed to the green pass in Italy, the main arguments of their discussions, and their characterization.

**Methods:**

We collected data from Telegram chats and analyzed the arguments and concerns that were raised by the users by using a mixed methods approach.

**Results:**

Most individuals opposing the green pass share antivaccine views, but doubts and concerns about vaccines are generally not among the arguments raised to oppose the green pass. Instead, the discussion revolves around the legal aspects and the definition of personal freedom. We explain the differences and similarities between antivaccine and anti–green pass discourses, and we discuss the ethical ramifications of our research, focusing on the use of Telegram chats as a social listening tool for public health.

**Conclusions:**

A large portion of individuals opposed to the green pass share antivaccine views. We suggest public health and political institutions to provide a legal explanation and a context for the use of the green pass, as well as to continue focusing on vaccine communication to inform vaccine-hesitant individuals. Further work is needed to define a consensual ethical framework for social listening for public health.

## Introduction

### Background

Since the beginning of large-scale vaccination campaigns against COVID-19, many countries have had to deal with the issue of vaccine hesitancy [1]. Already defined by the World Health Organization in 2019 as one of the major threats to global health [2], vaccine hesitancy has become even more relevant in the context of the COVID-19 pandemic [3]. In Israel, the first country that was able to ensure sufficient supplies of the Pfizer-BioNTech vaccine, the Ministry of Health swiftly started a vaccination campaign in late 2020. However, after covering health care staff, older adults, and vulnerable patients, the campaign reached a stagnation phase, as a relevant percentage of individuals were not willing to get vaccinated. After considering other forms of incentives [4], the Israeli Ministry of Health developed a new ad hoc strategy to increase the vaccination rate. According to this plan, vaccinated people would receive a special document that would allow them access to social and cultural events, national and international mobility, and exemption from quarantine. The declared aim of this document or the “green passport” was to encourage citizens to receive COVID-19 vaccinations while allowing some reopening of the economy [5]. The proposal for the Israeli green passport was passed on December 14, 2020 [4]; on January 27, 2021, the eHealth network of the European Commission started to develop a set of guidelines in order to implement a “EU Digital COVID certificate” system in Europe. On June 1, 2021, the European Union Gateway, that is, the backbone interconnecting national green pass systems in the European Union, went live [6]. On July 23, 2021 the Italian government passed Decree-Law 105, regulating the use of the green pass [7], already recognized and defined on April 22, 2021 by Decree-Law 52, article 9 [8].

Compared to other nudging strategies to tackle vaccine hesitancy, the green pass looks like a promising concept, as it incentivizes people to get vaccinated without imposing a decision; however, already in its first implementation in Israel, it generated some debate as it can be considered as a tool for discrimination based on someone’s vaccination status. Another argument often used by green pass critics regards privacy; when showing their green pass, people are de facto obliged to disclose health information—thus, their sensitive information—to third parties [5]. The adoption of the green pass strategy in Europe caused the very same debate and the very same arguments already seen in Israel. In many European countries, opposition movements started to form and grow, discussing the use of the green pass and organizing protests, sit-ins, and rallies [9]. In a time of physical distancing due to containment measures, many of these discussions were taking place online on social media and communication platforms. As popular social media platforms increasingly corrected their policies to decrease the flow of misinformation [10,11], people and organizations holding critical views about the green pass started to deplatform toward alternative social media channels, a phenomenon already seen and studied, mostly regarding the far-right and conservative world [12]. Notably, one of the most prominent destinations for deplatformed individuals and organizations has been Telegram.

Over the last few years, Telegram has become one of the most prominent instant messaging services. This success is due to a combination of 2 factors: on the one hand, end-to-end encryption [13] and an infrastructure distributed over several jurisdictions [14] makes it rather difficult to extract data from the system [15]. As stated on the official Telegram’s frequently asked questions, to this day, Telegram has “disclosed 0 bytes of user data to third parties, including governments” [14]. On the other hand, Telegram’s services go way beyond conventional instant messaging services: Telegram groups allow a maximum of 200,000 members and include advanced features such as unified history, instant search, replies, permissions, and moderation tools, making them outstanding tools for many-to-many discussions. In parallel, Telegram broadcast channels allow an unlimited number of followers, making them an appealing alternative to Twitter for one-to-many communication [14,16]. This combination of publicity, mobilization capabilities, and privacy provides a solution to the so-called “terrorists’ dilemma,” that is, the balancing security and outreach in choosing a web-based communication platform [17]. The use of Telegram among “no-green-pass groups” in Italy started to grow rapidly already in July 2021; as soon as the green pass was introduced, groups and individuals offering forged green passes for purchase started to exist [18] as well as groups organizing protests and rallies against the green pass [19].

### Aims

This study has 2 aims: (1) to study the discourse revolving around the opposition to the green pass and its use in Telegram chats by no-green-pass groups in Italy, with a focus on groups used by university students; and (2) to detail a novel approach to online social listening by using a combination of quantitative and qualitative approaches and to question its ethical aspects.

## Methods

### Ethical and Legal Considerations

As this study did not fall under the scope of the Swiss Human Research Act [20], authorization from the Cantonal Ethics Committee was not required. The messages analyzed in this study were retrieved from public chats using the “download history” function of Telegram Desktop. This qualifies the data as publicly available. According to the General Data Protection Regulation (GDPR) [21] article 6.1, data processing without explicit consent of data subjects is possible when protecting the interest of the data subject and when “necessary for the performance of a task carried out in the public interest.” Research falls in the category of public interest, but this criterion being very broad, it is important to weigh the public interest and benefits to the risk for the individuals, especially because the data set might contain special categories of personal data (ie, health, politics, or worldview-related data). Generally, the information detailed in article 14 of the GDPR should be provided to the data subjects individually, although this could be considered as a disproportionate effort, given the number of users involved in this study. However, one could argue that the necessary information could be provided in a general way through posting into those chats. Since either way this transparency might result in both a higher risk of reidentification and a serious impairment to the pursue of research, it could be argued that is against the public interest and should therefore be omitted. Articles 14.5 and 89 of the GDPR exempt from the provision of information to study participants where and insofar it would involve a disproportionate effort or render impossible or seriously impair the achievement of the objectives (ie, the research goals in the public interest). As specified in article 14.5.b, we took appropriate measures to protect the privacy of data subjects whose messages are included in our study: the JSON files retrieved from Telegram have been completely anonymized (removal of personal names and toponyms from the message text) and pseudonymized (replacement of the user ID with a pseudonym), the original data set has been destroyed, the analysis has been conducted on the anonymized version, the anonymized data set will be available upon request, and as the search of segments of text in the original chat would allow reidentification, the links to the chats will not be disclosed.

### Data Collection

Data were collected from 2 groups of chats. The first comprised no-green-pass groups of Italian universities (one in the north, one in the center, and one in the south) and generic no-green-pass groups. The second, our negative control, comprised groups discussing topics unrelated to COVID-19 or to the green pass, such as video games, parrot breeding, and other general topics. The selection of these control chats followed 3 criteria: the chats were active (at least 10 messages sent in the last week), they counted at least 200 users, and the main language used was Italian. We identified relevant chats and downloaded the message history as JSON files. We downloaded the JSON files containing the entire history of the said groups on September 9, 2021. The data collection is described in Multimedia Appendix 1.

Data were downloaded directly using the function “export chat history” of Telegram’s official desktop client. For this study, we downloaded only textual data. We parsed the JSON files into Pandas data frames. To protect the privacy of the users while still maintaining the possibility to track conversations in qualitative analysis, we combined anonymization and pseudonymization. Anonymization was performed by removing metadata from messages and by removing personal names and replacing them with [name] [22]. Similarly, every toponym was replaced with [place] [23]. Direct mentions of users in the text (eg, @thisuser) were searched and replaced with [username]. Surnames were not removed from messages. Since the chats were in an informal context, people did not refer to other members of the chat or to themselves using surnames. However, surnames are often used to refer to public figures or sources of information and thus represent a valuable component for the analysis.

### Analysis

For this project, first, we used a mixed methods approach, which involves the use of qualitative and quantitative data. Second, for the quantitative analysis, with a top-down approach, we defined a series of dictionaries relevant for the purpose of this study—each one containing regular expressions that belong to the same concept. Regex (Regular Expressions) allows the definition of fairly complex rules, able to reduce ambiguity, and capture precise concepts. As an example, the rule (tesser.\sverd.?|pass\sverd.?|certifica\w*\sverd.?) will fire on “tessera verde” (green pass) or “tessere verdi” (green passes) or “pass verde” (green pass) or “certificato verde” (green certificate) but not on “casa verde” (green house) or “verderame” (verdigris) or “tessera del cinema” (cinema card). The autocoding has a weight system: if only one rule from the dictionary fires, the autocode is assigned a weight of 1, if 2 rules fire, the weight will be 2 and so on. Autocodes can then be used to measure the prevalence of topics through the corpus to segment the quantitative analyses or as a starting point for the qualitative work. Third, we extracted the lemmas used in the corpus by using the Python package “spaCy” and its pretrained model for Italian [24]. This was performed on a large bag of words including every message in the corpus and by dividing messages by code. In the final step of the quantitative analysis, we performed a sentiment analysis [25], both on the entire corpus and on messages divided by code. The sentiment analysis was performed using the Python package “feel-it” [26], through which we calculated the probability of positive or negative sentiment for each message. We developed the analysis pipeline in Python; the code is structured in a JupyterLab notebook, available through Zenodo [27].

For the qualitative analysis, we generated a structured text file, annotated with pseudonymized speakers and codes resulting from the autocoding system. The file was then imported into MAXQDA for thematic analysis. The development of the regular expressions used for autocoding has been an iterative process. We ran the code several times, exploring the results, noting the gaps, and fine-tuning the regular expressions. The thematic analysis has been conducted by native Italian speakers on messages written in Italian; the text has been translated by the authors to be comprehensible to a wider audience but still as close as possible to the original. The original quotes in Italian and the categories/topics included in the analysis are provided as supplementary material (Multimedia Appendix 2 and Multimedia Appendix 3).

## Results

### Quantitative Results

#### Lemmas, Terms, and Rules: the No-Green-Pass Discourse Encompasses Legal Aspects, Actions, and Vaccine Skepticism

**Figure 1:**
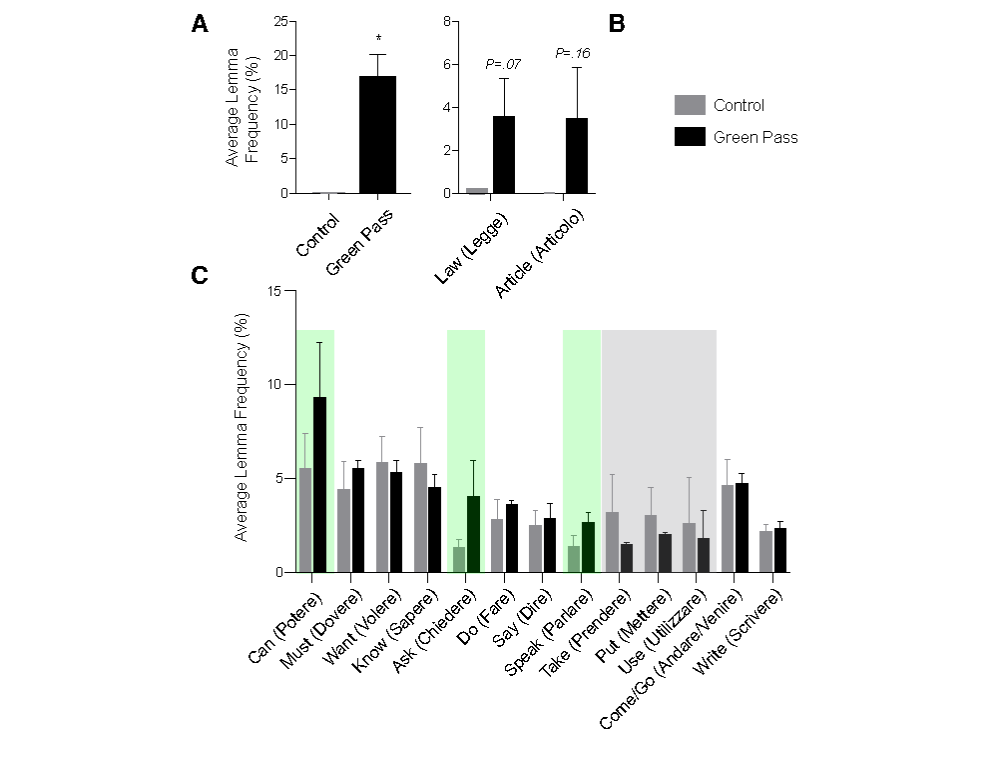
(A) Average lemma frequency (in percentage) in control versus green pass opposition chats. Average lemma frequency (in percentage) for green pass in control (grey bar) versus green pass opposition chats (black bar). (B) Average lemma frequency (in percentage) for legal terms in control chats (grey bars) when compared with green pass opposition chats (black bars), extracted from the 20 most frequently used words in the green pass opposition chats. (C) Average lemma frequency (in percentage) for action terms in control (grey bars) versus green pass opposition chats (black bars), extracted from the 20 most frequently used words in both control and green pass opposition chats. The green background highlights the most relevant action terms that are overrepresented in the green pass opposition chats, whereas the grey background highlights the most relevant action terms that are overrepresented in control chats. *P<.05, *t* test. Error bars represent standard error of the mean.

**Figure 2:**
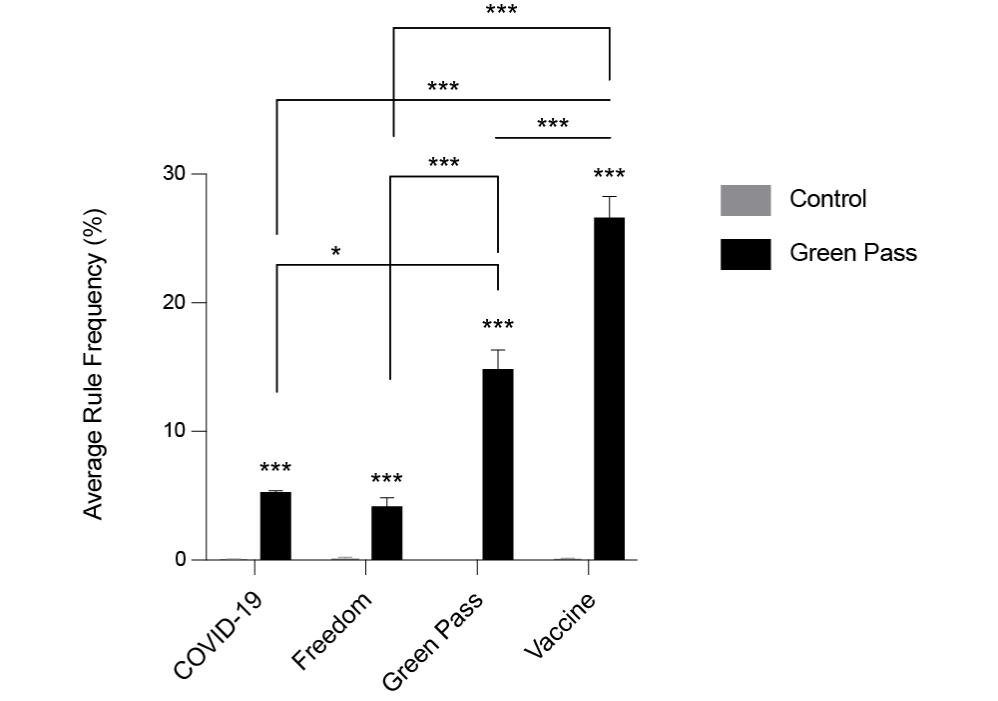
Average rule frequency (in percentage) in control versus green pass opposition chats. Average rule frequency for terms grouped under the rules “COVID-19,” “freedom,” “green pass,” and “vaccine” in control (grey bars) versus green pass opposition chats (black bars). *P<.05; ***P<.001, *t* test. Error bars represent standard error of the mean.

To understand the interests of individuals critical of the green pass, their arguments, and the opinions that shape their position in the debate, we quantified and analyzed the most frequently used lemmas in control chats (Multimedia Appendix 4)—discussing issues not related to green pass, vaccines, or COVID-19, and in chats focused on green pass opposition (Multimedia Appendix 5). As a positive control, we checked whether the lemmas “green” and “pass” were found to be among the most frequently used in green pass opposition chats when compared with control chats. As expected, “green” was the second most frequently used lemma in green pass opposition chats, and “pass” was the fourth most frequently used lemma (frequencies 9.2% and 7.5%, respectively). However, these lemmas were barely used in control chats (frequency of 0.02% for both lemmas). As expected, the average frequency of the 2 lemmas combined (“green” + “pass”) in green pass opposition chats was significantly higher than that in control chats (Figure 1A). Among the 20 most used lemmas in either control or green pass opposition chats, we identified 2 relevant categories of terms: legal terms and action terms. Legal terms included law (*legge*) and article (*articolo*). These terms were highly overrepresented in green pass opposition chats when compared with those in control chats (Figure 1B). Action lemmas included can (*potere*), must (*dovere*), want (*volere*), know (*sapere*), ask (*chiedere*), do (*fare*), say (*dire*), speak (*parlare*), take (*prendere*), put (*mettere*), use (*utilizzare*), come and go (*andare* and *venire*), and write (*scrivere*). Among these, we identified 3 lemmas to be relevant and underrepresented in green pass opposition chats: take (*prendere*), put (*mettere*), and use (*utilizzare*). Overrepresented lemmas were can (*potere*), ask (*chiedere*), and speak (*parlare*) (Figure 1C). Besides these lemmas, we were interested in understanding which rules were the most relevant in control chats, and, in addition to the rule “green pass,” we focused on the rules “COVID-19” and “vaccine” and “freedom.” With this analysis, we wanted to understand whether the legal lemmas that were overrepresented in green pass opposition chats were linked to a pronounced discussion about personal freedom, in connection with the discussion about the green pass and COVID-19 vaccines. All the abovementioned rules were represented with a higher frequency in green pass opposition chats when compared with those in control chats, and these differences were statistically significant (Figure 2). As expected, the rule for “green pass” fired very frequently in green pass opposition chats and more frequently than the rules “COVID-19” and “freedom.” Surprisingly, however, the rule “vaccine” was the most frequently used in green pass opposition chats, more so than the rule “green pass,” indicating that among green pass critics, even when the discussion revolves around legal aspects connected to personal freedom, skepticism toward vaccines likely remains as the predominant reason to oppose the green pass.

#### “No-Green-Pass” Individuals Have a Negative Sentiment Toward Green Pass and Vaccines

**Figure 3:**
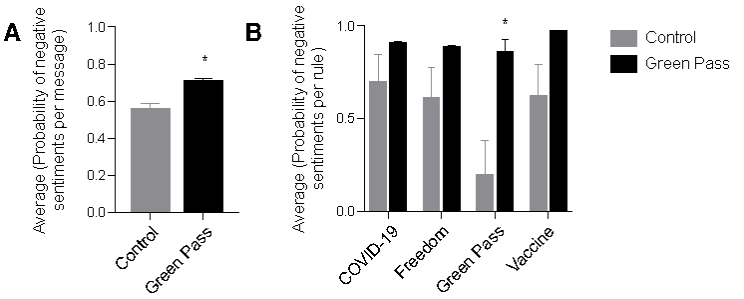
Sentiment analysis in control versus green pass opposition chats. Average probability of negative sentiment in messages published in control (grey bar) versus green pass opposition (black bar) chats. (A) Average probability of negative sentiment per rule in control (grey bars) versus green pass opposition chats (black bars). The following rules are taken into consideration: “COVID-19,” “Freedom,” “Green Pass,” and “Vaccine” (B) 0 indicates the maximum likelihood for an average message to display positive sentiment, whereas 1 indicates the maximum likelihood for an average message to display negative sentiment. *P<.05, *t* test. Error bars represent standard error of the mean.

After having identified the predominant themes associated with anti–green pass discourse, we analyzed whether such a discourse is associated with a higher probability of negative sentiments. By defining the likelihood of negative sentiment for each message, we averaged the sentiment for each chat and finally across chats within the same category. As expected, the average likelihood of negative sentiment was significantly higher in green pass opposition chats when compared to that in control chats, with a probability of 0.70 and 0.55, respectively (Figure 3A). In addition, we calculated the average probability of negative sentiment associated with the rules “COVID-19,” “freedom,” “green pass,” “vaccine,” and determined that for all these rules, messages depicting negativity were overrepresented in green pass opposition chats when compared with those in control chats (Figure 3B). This effect was significant for the rule “green pass,” which can serve as a positive control, indicating that green pass critics are, in fact, assessing the issue with negative sentiment, when compared with people that do not necessarily oppose its introduction and use. Of particular interest, messages related to the rule "vaccine" had a 96.26% probability to depict negative sentiment, a particularly high probability also when compared with negativity for "COVID-19", "freedom", and "green pass" in green pass opposition chats (90%, 88%, and 85%, respectively), thus providing strength to the hypothesis that vaccine skepticism is the primary reason to oppose the green pass.

#### Rules and Lemma Frequency: Interplay Between Vaccines and Green Pass

To further understand the relationship among the topics “green pass,” “vaccine,” “freedom,” and “COVID-19,” we analyzed the most frequently used lemmas when the discussion was on one of such topics (as determined using the associated rules). For the rule “COVID-19,” the lemmas “green,” pass,” and “vaccine” were among the most used (Figure S1A in Multimedia Appendix 6). For the rule “freedom,” as expected, lemmas associated with legal terms were overrepresented as well as “green” and “pass” (Figure S1B in Multimedia Appendix 6). For the term “green pass,” we could not identify “vaccine” among the most relevant and used lemmas, although we identified lemmas associated with legal terms, including “freedom,” “law,” “article,” “can,” and “must” (Figure S1C in Multimedia Appendix 6). Instead, for the rule “vaccine,” we could identify “green” and “pass” among the most relevant and significant lemmas (Figure S1D in Multimedia Appendix 6). As our previous results indicate, although our analysis was focused on green pass opposition chats, vaccines constituted a widely discussed topic, which even dominated the discussion about the green pass. In line with our previous observations, here we show that green pass discussion takes place when vaccines are being discussed but not vice versa. This might suggest that green pass critics tend to share antivaccine views but do not wish their argumentations against the green pass to revolve around their antivaccine views. Rather, they prefer to support their position by discussing limitations to personal freedom and advancing legal considerations.

### Qualitative Results

#### Green Pass and Vaccines

The qualitative analysis supports the findings described in our quantitative analysis. Although our analysis was focused on chats discussing the green pass, users often started debating about related topics, including the risk-benefit profile of COVID-19 vaccines, their efficacy, and their use. Of note, moderators often asked participants to stay on topic and avoid discussing these parallel issues. There were 2 main reasons: one was to avoid conflict, as a (small) fraction of individuals who positioned themselves as opposed to the green pass were for vaccinations; the other was to avoid floods of misinformation, which could discredit what the moderators perceived as a much needed debate. The quotes below are shown by the subcorpus (university group or generic group, and the position refers to the line in the subcorpus).

…On the other hand, it is a big mistake to take a stance on vaccines. Those who want to do so should do so. The point is only to be against this limitation of freedom and many vaccinated people are against the green pass. Do not introduce divisive or extremist elements that vote the initiative down.

University, south, position 742

Users themselves are very aware of how hard it is to discuss the green pass without discussing the reasons for which it is needed.

…how can one ignore the vaccine issue if it is literally the main option for getting a pass?

University, north, position 6693

As stated above and as noted in the quantitative analysis, these no-green-pass chats have de facto been a proxy to discuss vaccines. Users know the green pass was introduced as a nudging measure to avoid mandatory vaccination. Nevertheless, they do not perceive this strategy in a positive way. Even if there are other ways to receive a valid green pass (ie, recovery from COVID-19 or testing), vaccination is the most obvious and less burdensome one. Users perceive this as a cunning imposition, which possibly makes them even angrier than mandated vaccines.

…I am against the green pass because I see it as a coercive and hypocritical tool put in place by the government because if they saw the vaccine as a safe way, they should have the consistency to make it compulsory and instead they don't bother to do so.

University, south, position 1807

…the green pass is a way of circumventing compulsory vaccination. The green pass is an “incentive,” said to be very soft, but in fact it is a compulsory requirement.

University, center, position 14716-14718

Even though, as discussed above, moderators would prefer to disentangle the discussion about the green pass from the topic of vaccinations, pointing out that even someone who is vaccinated could hold no-green-pass positions, most of the users shared common critical beliefs about vaccines.

…It is becoming increasingly clear from the scientific literature that (1) There are very effective treatments for COVID that indicate that vaccines are not at all essential. (2) Vaccines often have serious short-, medium- and long-term side effects, there is a well-founded fear that they could induce serious pathologies (tumors, autoimmune and degenerative diseases, sterility...) and they are still at the experimental stage. (3) Vaccines facilitate the development of variants, many of which are particularly virulent, and should not be carried out during epidemics, let alone pandemics. (4) Vaccines do not absolutely protect against COVID as they are said to do, ie, those vaccinated may become infected and may in turn infect others... so they should not have a Green pass unless they too are swabbed.

University, center, position 3572-3579

Users are especially afraid of the possible side effects. This narrative proposes that the vaccine is worse than the disease it is meant to prevent.

…who can guarantee that I will not have serious effects as a result of the vaccine, which could harm my future? Who will compensate me for any damage?

University, north, position 25293-25294

…Statistics show that the number of deaths due to COVID is the same as the number of deaths due to the vaccine, only that the number of deaths due to COVID is much overestimated (the number also includes deaths due to other causes but catalogued as COVID because they are positive to the test), while deaths due to the vaccine (not to mention cases of serious adverse effects) are much underestimated because only passive surveillance is done, and poorly.

University, center, position 15682-15688

Moreover, according to several users, there is no evidence that vaccines work. They do not prevent the spread of the disease and are less effective and more burdensome than alternative therapies to reduce the symptoms (the most quoted are hydroxychloroquine, cortisone, heparin, ivermectin, nonsteroidal anti-inflammatory drug, and hyperimmune plasma transfusions).

…We must rebel, this vaccine is a gene therapy with no guarantee that it will work. Vaccinated people are just as infectious as unvaccinated people, it is clear that this vaccine does not protect against COVID.

University, north, position 2612

…It is written in all official documents of the pharmaceutical companies and the WHO that there is no evidence that vaccination will stop the spread of the virus.

University, north, position 3385

Finally, some users suggested that vaccines could be part of a bigger scheme, again orchestrated by governments and covert powers, possibly aiming to reduce the world’s population.

…Overpopulation, they have been saying this for years, and the Vax in my opinion serves to solve that problem, not COVID.

University, south, position 2343

…Their aim is to manipulate human beings by injecting them with a serum containing graphene, which can react with certain frequencies and modify the behavior of cells. By changing the behavior of cells, you can change the behavior of human beings.

Generic, position 72471

#### Beyond Vaccines: Green Pass, Legal Aspects, and Personal Freedom

Despite vaccines being the predominant topic in these chats, the majority of individuals did not make use of arguments related to vaccines, including conspiracy theories about vaccines, to justify their opposition to the green pass. Rather, they claimed the green pass was an illegal measure and it is discriminatory.

…it is clear that the green pass is an instrument of political discrimination that has no relation to the actual health status.

University, center, position 3572-3579

…The green pass is clearly unconstitutional and discriminatory in nature and is a purely political instrument as it has no scientific basis; the report linked before is very clear about it, then they do not make it mandatory by law otherwise they would be obliged to compensate those who died of the vaccine.

University, center, position 7520-7522

In some circumstances, users alluded to conspiracy theories according to which the green pass is an element of a bigger plan put in place either by governments or by covert powers to achieve other ends, usually the institution of a totalitarian regime.

…Do you still have to realize that even if the Regime decides to withdraw the COVID PASS, to let you go back to work, you have already become citizens of a totalitarian Regime? Citizens of a lousy Regime based on lies, on the progressive elimination of freedoms, on the violent suppression of dissent?

Generic, position 2127

Many users believed the green pass was a serious limitation of personal freedom. This argument was developed following 3 main threads in order of importance: jurisprudential, consequentialist, and deontological. On the jurisprudential side, users appeal mostly to the Italian constitution (articles 13 and 120), to law 196/2003 (personal data protection code), and to the Oviedo convention.

…the “green pass” cannot be checked because it is discriminatory, prejudicial to privacy and violates the following articles of law: - Art. 187 of the TULPS Regulation: a commercial operator is obliged to welcome in his business any person, without discrimination, under penalty of a fine up to €3000,00.- Privacy Law: no one can force us to provide information about our health conditions.- Art. 120 Italian Constitution: no one can limit the freedom of movement of the individual in the territory of the Italian republic. - Art. 13 Italian Constitution: no one may restrict personal freedom without a provision of the Judicial Authority on facts concerning the individual.

Generic, position 3448

…Add that we will respect all the anti-COVID security measures (social distancing, hygiene, mask). With regard to the reference to laws and treaties, don't we want to mention the convention on human rights, the Oviedo treaty and the Supreme Court ruling stating that the health of the individual cannot be sacrificed for the sake of collective health? don't we want to mention the principle of self-determination?

University, center, position 395-397

On the consequentialist side, users tend to fall into a slippery slope fallacy. In their view, the green pass system will necessarily lead to a system of capillary social control, repression of dissent, and loss of critical thinking capabilities—a system that is clearly undesirable and immoral; therefore, the green pass is undesirable and immoral as well.

…Look at the Chinese social score system to understand the crazy direction of these actions, typical of dictatorial systems and not of advanced democracies.

University, south, position 3755

…By now I think these people are lobotomized and probably don't even know the word FREEDOM.

University, south, position 1255

A minority of users tried to build a deontological argument, balancing values such as freedom and life. Their conclusion was that life and freedom have equal importance; hence, it is unjust to protect life by limiting freedom.

…If the answer to the question is that life is more important than liberty, then all the liberticidal laws made so far are justifiable and I would say almost fair, I can also understand why the green pass, a blatantly discriminatory law, is considered fair by many.

…If the answer to the question is freedom, it is clear that everything that has been done so far is considered a mistake regardless of whether a particular law was made to save lives.

…We come to the last answer, the most balanced one for me at least, that life and freedom are of equal importance. In view of this answer, it is clear that taking precautions to limit contagion and death is right and proper, so limitations will be inevitable (such as social distancing, masks indoors, limiting seating etc.), but at the same time it is important to preserve the freedoms of all citizens.

University, center, position 14996-15012

The 3 arguments converge on a single conclusion: the green pass and the system of control it creates are either seen as tools in the hands of dictators or as preparatory tools to gather power.

…What kind of disgusting nightmare do we want to bequeath to our children? A Health Regime? A Regime that brutalizes the minds and bodies of its citizens on a daily basis? Enough! Rebel!

Generic, position 2127

…We are living in a health dictatorship and political authoritarianism that must be opposed. I wonder if a general acting as a commissioner who comes out with absurd words about wanting to flush out the 'unvaccinated' house by house? These people must leave the government. We must demand to go to the polls again.

University, center, position 5904-5906

#### Action Plan

Leveraging on this understanding of freedom, users perceive a clear duty to react. The first and foremost action is understanding who the real enemy is, that is, not the virus, nor the people who get vaccinated or obey the regime. The real enemies are the political system and the political representatives who allowed this to happen.

…It is a political issue everywhere. If we understand this we know who we have to fight, and for sure it’s not a virus.

University, north, position 20112

In the university groups, users discussed a lot about communication strategies that would allow them to be credible, also because they are aware that their groups might be studied. The most important points were regarding avoiding defusing topics (ie, conspiracy theories) and focusing on self-determination. Again, coherently with our quantitative findings, the main issue appeared to be the vaccine, for which the green pass was just a proxy.

…we have been able to ascertain the intense doxing activity also of Telegram groups. In short, now that membership is growing, we need a minimum of 'art of war' (or rather strategy, just to avoid accusations of terrorism).

University, north, position 20233

…no disquisitions that go beyond the topic to be defended, such as the existence or nonexistence of the virus, the no-pro vax diatribe, the Davis forum, depopulation, mass experimentation, variants, damage, etc. These are all topics on which one has burnt the candle at the stake. These are all topics on which the authoritativeness of many prominent figures has been burned, since they easily fall under the so to speak 'defusing' labels (conspiracist, degree obtained on Google, no Mask, no vax, no test, denialist).

University, north, position 3607

Lastly, many users considered protests as valid strategic options to make their voices heard. The options they considered ranged from flash mobs to general strikes, to occupations of the parliament.

…Shall we make a flash mob where all the unvaccinated all go in at the same time where they can't? Maybe running so that we are sweaty (so they are afraid to touch us) maybe with a hat that says “the Jew rebels”

Generic, position 1007

…You will sign in front of the incredulous eyes of your employer your declaration of nonviolent struggle. Your declaration of an all-out general strike. Full stop. Nothing else is needed. There will be 100,000 of us, and we will block Italy, offices, services, production. We will pull the plug of this infamous regime.

Generic, position 2127

### Summary: Explaining Green Pass Opposition Without Involving Vaccines

Among those opposing the introduction of the green pass, especially among university students, only a few were in favor of vaccinations and those in favor of freedom of choice were typically hesitant about vaccines. The “being aware” antivaccine discourse has been typically dismissed by a large fraction of the Italian society and by the political class as conspiratorial in nature and not worth considering. Anti–green pass antivaccine supporters have oriented themselves toward different argumentations to defend their positions, revolving around legal aspects related to the concept of personal freedom. Our considerations are well summarized by the following message.

…The main argument must continue to be that one must be able to refuse an injection, whatever it may be. The body is mine and I decide. And if you were to be convinced that the serum prevents x% of the infection (as some try to suggest), would our whole battle fall apart? I certainly hope it’s not the case.

University, north, position 24367

The battle is fought on grounds different from that of vaccines, but vaccines are what this battle is for.

### Other Aspects

#### COVID-19

In the no-green-pass corpus, 2 main positions about COVID-19 emerged. According to the first, COVID-19 exists but is much less dangerous than what it is communicated by the mainstream media.

…In addition, in response to the pathetic provocation, I would like to point out that 99% of COVID deaths are of over-80s with multiple pathologies.

University, center, position 2199-2202

…COVID exists but you can't stop the world because of it. It's a fucking flu, especially for young people. Many more people have died of the flu and it has never been talked about.

University, north, position 2864

According to the second dominant position, COVID-19 does not exist and is yet another element of a bigger plan conceived to limit personal freedom and eradicate free thinking through fear.

…Do you realize that you're talking about a virus that nobody anywhere in the world can prove exists?

University, north, position 1328

…The virus has never been isolated or purified.

University, north, position 6509

At the junction of these 2 narratives, COVID-19 would be a strategy to pursue other means.

…the virus is just a means to achieve other goals that have nothing to do with health protection.

University, center, position 8092-8095

#### Expertise

If COVID-19 does not exist or is not particularly dangerous, then the need for measures such as the green pass would be unfounded. These beliefs are supported by a wide network of experts, which according to users are brave free thinkers who are not afraid of speaking their mind and standing against these covert powers.

…In addition, the most important doctor we have in Italy, Dr Remuzzi with H index 189, has long since drawn up an approved treatment protocol. Go to the website of the [name] Negri Institute and find out more. Dr Scoglio, candidate for the 2018 Nobel Prize, should also be considered.

University, center, position 14640-14643

…COVID can be treated at home, with medication. There is a group of volunteer doctors who do just that. “Terapie domiciliari COVID,” a very popular Facebook group.

University, south, position 1974

…Listen also to Dr Citro, Dr [name] Montanari, Dr Bolgan, to what they say. [Authorities] forced people to get vaccinated with fear, and blackmailed young people with the green pass. There are many adverse reactions and they don't tell you that, so resist for your own good.

University, center, position 4198-4200

…The mask does not protect against viruses. Instead, it creates colonies of bacteria that you breathe in, as well as other filth that I won't tell you about, not to look like a conspiracy theorist. My colleague's comments on Dr Gatti are right. A great nanopathologist.

University, north, position 742

…According to Dr Delgado, it is not a virus that causes the disease. I will explain this when we meet.

University, north, position 3485

#### Preferred Measures

Among those who believe that COVID-19 is actually an issue to be contained, some try to delineate alternatives to the green pass. These include the use of masks, social distancing, tests, and dual teaching (both in presence and online).

…Exactly, you must respect all the rules to prevent contagion and therefore masks and distancing.

University, south, position 1467

…if we really want to be sure that the virus does not spread in the university, shouldn't the swab be used for everyone who enters the university, as it is the only instrument with a high percentage of detection of the virus?

University, north, position 25297

…However, I would like to see mixed teaching, both face-to-face and online, at least in the first semester so as not to increase the risk of infection and to allow everyone to get vaccinated. The situation in [place], with transport and everything, means that the risk of contagion is too high, even for those who are vaccinated and may be carriers. I don't feel like taking the responsibility of walking around in [place], even if I'm vaccinated, and putting other people's life at risk.

University, center, position 2095-2102

However, in the same groups, there is a strong critique of the dehumanization caused by online teaching, and tests are perceived as burdensome (economically and physically) and as unfair.

…In spite of the effort to reach the university, it is not real university what you do online. [The real one is] made of people, looks, REAL dialogues; it is precisely the effort and the time spent to go to the university that sanctions its founding and formative value. Distance learning is not an appropriate cultural medium.

University, north, position 19204

…the test becomes an economically limiting tool for the individual, since university students are not guaranteed free access to this service at all, which puts an economic burden on those who choose not to vaccinate.

University, north, position 25298

#### Antitest and Antimask Positions

Although more testing and systematic use of masks are sometimes suggested as a preferred protection strategy, many users have concerns about both. Some users think that tests and masks do not work; some think tests are dangerous as while collecting a mucus sample, it is possible to damage the brain; and some believe that masks are dangerous as they create bacterial colonies.

…I still don't understand... (it's rhetorical and sarcastic) why for the most contagious virus that spreads with a single droplet - with aerosol even, in the air... you have to pierce all the way to the encephalic barrier and up to the pineal gland? Maybe because otherwise you don't assimilate graphene oxide & who knows what else? Vets have long used nasal vaccination. Ps. There have been cases of rhinoliquorrhoea, ie, loss of cerebrospinal fluid, dizziness, abnormal migraines, etc, but of course, as with everything else, everything is covered up and minimized.

University, north, position 11697-11698

…The mask does not protect against viruses. Instead, it creates colonies of bacteria that you breathe in, as well as other filth that I won't tell you about, not to look like a conspiracy theorist. My colleague's comments on Dr Gatti are right. A great nanopathologist.

University, north, position 742

#### Reliance on Anecdotal Evidence

Users often bring information to support their claims. Sometimes, they provide links to blog posts but seldom to scientific studies or to statistical analyses. Sometimes, they engage with such information critically; sometimes they do not. Of note, stories based on anecdotes and personal narratives tend not to be questioned.

…My grandfather died with COVID. We followed what the doctors said about treatment at home for my grandmother. She survived. My grandfather wanted to follow the standard procedure instead. 2 weeks worsening. Intensive care and death.

University, center, position 13863-13866

…I spoke to a doctor from [place]. Do you know what they do to make it look like only the unvaccinated are in the ICU? When COVID patients come in, even those vaccinated with two doses, they have orders to move the vaccinated to other wards and leave the unvaccinated in the ICU.

University, north, position 24524

## Discussion

### Vaccine Skepticism and Public Health Recommendations

Our analysis clearly shows how the green pass has become a proxy and a catalyzer for vaccine skepticism. Especially during this time, people and politicians supportive of vaccines strongly oppose vaccine skepticism or denialism. The discussion about the dangers of vaccines as well as the conspiracy theories and the misinformation in general are not considered relevant and are silenced since these positions are not backed up by scientific evidence. Antivaccine supporters have come to learn that shifting their focus on the green pass allows them to bring new arguments, which are more likely to be heard, to indirectly bring arguments against the use of vaccines. In fact, questioning the validity of the green pass rather than that of vaccines is seen as less socially problematic, albeit it remains strictly connected to the discussion about vaccines. In practice, the green pass has become the fig leaf of the antivaccine movement. That said, it is also important to note that tensions and diverging narratives exist, even within the groups under analysis. As our results show, moderate positions (ie, COVID-19 is an issue, but the green pass is not an appropriate measure) coexist with conspiracy theories (ie, COVID-19 does not exist and COVID-19, vaccines, and the green pass are part of a bigger plan). De facto, opposition to the green pass is what glues together these opinions and attitudes. This opposition is often justified on the grounds of a naïve idea of freedom, conceptualized in a jurisprudential, consequentialist, or deontological form. Based on our findings, we believe that it is possible to trace some recommendations for public health authorities and political institutions engaging with communication on these topics:

1. Acknowledge the doubts of individuals opposed to the green pass without dismissing their opinions and arguments as ramblings.
2. Disambiguate the purpose of the green pass: it should be made clear that it is a tool intended to incentivize vaccinations and thus to protect people—not only to people who cannot get vaccinated but also to protect everybody’s personal freedom (ie, those who are not willing to risk to contract the virus but still desire to enjoy a meal in a restaurant, watch a theater play, or a football match in a stadium, etc). We see this discussion as a reminiscent of the long-standing debate about smoking in closed environments.
3. Since freedom is an important topic, counteract the models of freedom in which the opposition to the green pass is grounded, offering alternatives, for example, Rawls’ “greatest equal liberty” principle [28], according to which each person should be given the most extensive basic freedoms that are compatible with another person’s freedom.
4. Clarify the legal basis of the green pass, explaining how it is founded and regulated in existing jurisprudence, and how its scope and application is defined and limited by the contingency of the pandemic. It is necessary to explain why it has a specific “expiry date” and under which circumstances and for how long people should expect these measures to be in place.
5. Keep informing about vaccines, with a specific focus on transparency and risk-benefit balance. In this context, complement as much as possible narratives based on data and scientific evidence with personal narratives (still backed up by science), as they are easier to relate to and can be more effective [29].

### Ethical Considerations and Recommendations

#### A Plea for Active Social Listening

Communication is a key component of human life. The ability to communicate privately with others can be understood as an expression of the right to privacy. Privacy, in turn, is not a luxury that can be easily overridden by other seemingly more urgent or more important needs. Rather, it is a fundamental human right recognized by the United Nations Declaration of Human Rights and many other international and national treaties. The COVID-19 pandemic has presented us with tricky dilemmas regarding the protection of both privacy and public health. Although there is no doubt about the need for the effective management of the pandemic, concerns have been voiced that “measures taken to control the spread of COVID-19 have negatively impacted the enjoyment of the right to privacy and other human rights” [30]. These concerns become even more acute when measures are coupled with artificial intelligence technology that can enhance not only analysis and forecasting but also the ability to effectively target the behavior of groups and individuals [31]. The key ethical question is therefore how effective communication and management during important public health crises such as pandemics is possible without undermining privacy as a human right.

Telegram grants end-to-end encryption, and encrypted communication might grant a sense of safety to users. In fact, owing to this perceived safety, often it is chosen for illegal activities as it happened for the sale of false green passes [18]. However, when a curious user acquires access to the group, either directly or with social engineering techniques, he has access to the entire history of the chat, no matter the encryption. It is worth noting that similar or related groups often cross-share messages; when a message is shared, it incorporates a link to the original chat where it was posted. Thus, by scraping chats for “t.me” links, it is rather simple to obtain access to related groups. Finally, it is important to mention that often these groups use bots offering more advanced moderation features, for example, silencing a user for a specified amount of time. As bots come to users as black boxes, it would not be difficult to load them with malicious features, for example, sending the links of the chats where they are used if specific rules fire. Even when users do not use their name and surname as their username, still there are many possible strategies for reidentification. Users might share emails, locations, and even pictures. Crossing this information and identifying a person is just a matter of amount of data, time, and commitment.

Having proven that the approach and the techniques detailed in this paper can provide useful and deep insight on critical topics debated in Telegram groups, we still tend to think that these techniques should not be applied broadly for social listening. We live in a time in which societies are already experiencing a progressive loss of trust, and techniques of passive social listening—intended as collecting information from digital communities without engaging with them—can only worsen the situation. Passive social listening, as detailed in this paper, is incredibly powerful, as it can extensively and rapidly map communities, measure their discussions, and potentially help predicting protests and violent actions. Active social listening—intended as actively asking people their opinion on delicate topics such as vaccine distribution strategies or safety measures—is slower and less comprehensive, as it depends on creating efficient bidirectional interfaces between the public and authorities. However, it has a big advantage: it can build trust rather than undermine it further. Engaging directly with communities by offering concerned people the possibility to voice their worries can create a sense of not only being listened to but also of being heard, recognized, and valued. A recent example of an active social listening tool is PubliCo [32], a web-based platform that collects data on public perception of the pandemic and its management. Following a participatory citizen science approach, it invites users not only to provide data but also to suggest new survey items or to research the database with queries of their particular interest [33].

#### Transparency and Recommendations

The software and the procedure we developed are subject to the dual-use problem. In nondemocratic regimes, they could be used not only to map and understand dissent but to eradicate it. Our decision to share it is motivated by 3 reasons: first, science should be open and transparent in its objectives, means, and methods, not only in its findings. Second, as Steven Levy noted, “If you don’t have access to the information you need to improve things, how can you fix them?“ [34]. Pavel Durov, Telegram’s founder, stated that “Telegram must continue to serve the world as an example of a tech company that strives for perfection and integrity” [35]. If Telegram wants to stay true to that claim, the company needs to know how a characteristic of their software can be exploited as a vulnerability compromising users’ privacy. Third, if a nondemocratic regime would want to develop a similar system, it could do it anyways unless this vulnerability is fixed.

### Limitations

As we collected our data from public Telegram groups, our sampling is not representative of the general anti–green pass population. We do not have any information about the magnitude of the phenomenon nor do we have demographic variables to stratify the analysis. However, the sample is relevant for the scope of this study and we can characterize why and how these groups oppose the green pass by drawing reliable conclusions and outlining possible approach strategies. Our approach to thematic analysis departs from standards: in thematic analysis, data should be disassembled and reassembled in a different shape to reveal its themes and patterns [36,37] with a bottom-up approach to coding. Codes should emerge during the analysis to capture emerging and unforeseen phenomena, which contrasts with the very notion of autocoding that we employed. To mitigate this, we adopted an iterative process with continuous testing, analysis, and expansion of the rules. Still, we believe autocoding is a good compromise to map the content of large volumes of data in reasonable time.

### Conclusion

Through our social listening analysis on Telegram chats, we conclude that a large fraction of individuals opposed to the green pass share antivaccine views. We also show they generally do not argue their opposition to the green pass with antivaccine rhetoric but rather focus on the legal aspects and limitations of personal freedom. We suggest that public health and political institutions provide a legal explanation and a context for the use of the green pass as well as to continue focusing on vaccine communication to inform hesitant individuals. Finally, we point to the ethical ramifications of our research and propose ways to ensure that social listening analysis is transparent and ethically sound. Further work is needed to define a consensual ethical framework for social listening for public health.

## Appendix

Multimedia Appendix 1 Data collection: composition of the groups, users, and number of messages. The table lists the category to which the groups belong (no-green-pass or control), the description of the group, the number of users, and the number of messages in the group.

Multimedia Appendix 2 Original quotes in Italian included in the qualitative analysis.

Multimedia Appendix 3 Categories and topics included in the qualitative analysis.

Multimedia Appendix 4 The 20 most frequently used lemmas, symbols, or expressions in control chats. The table lists the most frequently used lemmas, symbols, or expressions (in percentage) on average across each individual control chat (n=5).

Multimedia Appendix 5 The 20 most used lemmas, symbols, or expressions in green pass opposition chats. The table lists the most frequently used lemmas, symbols, or expressions (in percentage) on average across each individual green opposition chat (n=5).

Multimedia Appendix 6 Average lemma frequency (in percentage) per rule in control versus green pass opposition chats. Average lemma frequency in percentage for the 10 most frequent lemmas in green pass opposition chats (black bars), compared with their relative frequency in control chats (grey bars), extracted from messages scoring a positive value for at least one of the rules.

## Abbreviations

GDPR: General Data Protection Regulation
