# Supplementary material for: Concerns Around Opposition to the Green Pass in Italy: Social Listening Analysis by Using a Mixed Methods Approach
Source: J Med Internet Res. 2022 Feb 16;24(2):e34385. doi: 10.2196/34385 (PMC8852653; doi:10.2196/34385)
Supplement: Multimedia Appendix 1 [file jmir_v24i2e34385_app1.docx]

#### Multimedia appendix 1

| **Category** | **Group description** | **n of users** | **n of messages** |
| --- | --- | --- | --- |
| no green pass | university, north | 1770 | 7356 |
|  | university, center | 5168 | 10464 |
|  | university, south | 479 | 1879 |
|  | generic | 12295 | 33707 |
|  | *Total* | *19712* | *53406* |
|  |  |  |  |
| control | parrots | 296 | 48494 |
|  | videogames | 750 | 43322 |
|  | generic | 294 | 10588 |
|  | generic | 210 | 1453 |
|  | generic | 218 | 21611 |
|  | *Total* | *1768* | *125468* |
