# Supplementary material for: Concerns Around Opposition to the Green Pass in Italy: Social Listening Analysis by Using a Mixed Methods Approach
Source: J Med Internet Res. 2022 Feb 16;24(2):e34385. doi: 10.2196/34385 (PMC8852653; doi:10.2196/34385)
Supplement: Multimedia Appendix 2 [file jmir_v24i2e34385_app2.docx]

Supplementary material: original text in Italian

### Green pass and vaccines

Sul vaccino invece è un grave errore prendere posizione. Chi lo vuole fare lo faccia. Il punto è solo essere contrari a questa limitazione di libertà e molti vaccinati sono contrari al green pass. Non introducete elementi divisivi o di estremismo che votano al fallimento l’iniziativa (university, south, Pos. 742)

come si può ignorare la questione vaccino se è letteralmente l’opzione principale che permette di ottenere un pass? (university, north, Pos. 6693)

Sono contrario al green pass perché lo vedo uno strumento coercitivo e ipocrita messo in atto dal governo in quanto esso, se vedesse nel vaccino una strada sicura da seguire dovrebbe avere la coerenza di renderlo obbligatorio e invece non si prende la briga di farlo (university, south, Pos. 1807)

il greenpass è un modo per raggirare l'obbligatorietà che non può essere messa. Il Green pass è un "incentivo" detto molto soft, ma di fatto appunto un obbligo messo a tavolino. (university, center, Pos. 14716 – 14718)

Dalla letteratura scientifica risulta sempre più chiaramente che: 1) Esistono cure molto efficaci oer il Covid che indicano che i vaccini non sono affatto indispensabili. 2) i vaccini hanno spesso gravi effetti collaterali a breve, medio, e a lungo termine, esiste il fondato timore che potrebbero indurre gravi patologie (tumori, malattie autoimmunitarie e degenerative, sterilità…) e sono tutt’ora in piena fase sperimentale. 3) i vaccini facilitano lo sviluppo di varianti, molte delle quali particolarmente virulente, e non andrebbero eseguiti in fase epidemica e tantomeno pandemica. 4) i vaccini non proteggono in modo assoluto dal Covid come viene detto, ovvero i vaccinati si possono infettare e possono a loro volta contagiare… per cui non dovrebbero avere il Green pass se non facendo anche loro il tampone… (university, center, Pos. 3572 – 3579

chi mi garantisce che a causa della somministrazione del vaccino io non abbia degli effetti gravi che potrebbero ledere il mio futuro? Chi mi risarcirebbe di eventuali danni? (university, north, Pos. 25293-25294)

Le statistiche evidenziano che il numero dei morti per Covid e’ uguale a quello dei morti da vaccino soltanto che il numero dei morti per Covid e’ di molto sovrastimato (il numero comprende anche i decessi per altre cause ma catalogati come Covid poiche’ tampone positivi) mentre i morti da vaccino (senza parlare dei casi di effetti avversi gravi) sono molto sottostimati in quanto viene fatta poco e male solo la vigilanza passiva. (university, center, Pos. 15682 – 15688)

Bisogna ribellarsi, questo vaccino é una terapia genica senza alcuna garanzia di funzionamento. I vaccinati sono infettivi come i non vaccinati, é evidente che questo vaccino non protegge dal COVID. (university, north, Pos. 2612)

É scritto in tutti i documenti ufficiali delle case farmaceutiche e dell’OMS che non c’é alcuna evidenza che la vaccinazione fermerá la diffusione del virus (university, north, Pos. 3385)

Sovrappopolazione, c'è lo dicono da anni, ed il vax secondo me serve a risolvere quel problema altro che covid... (university, south, Pos. 2343)

Il loro obiettivo è arrivare alla manipolazione dell'essere umano iniettando in questo un siero che contiene grafene che poi con certe frequenze può reagire e modificare il comportamento delle cellule. Modificando il comportento delle cellule puoi modificare quello dell'essere umano (generic, Pos. 72471)

### Beyond vaccines: Green pass, legal aspects and personal freedom

È EVIDENTE CHE IL GREEN PASS È UNO STRUMENTO DI DISCRIMINAZIONE POLITICA CHE NON HA ALCUNA RELAZIONE CON L’EFFETTIVO STATO DI SALUTE… (university, center, Pos. 3572 – 3579)

Il green pass è palesemente incostituzionale e di natura discriminatorio ed è esclusivamente politico dato che non ha alcuna base scientifica visto che il rapporto prima enunciato è molto chiaro a riguardo,poi non lo rendono obbligatorio per legge altrimenti sarebbero obbligati a risarcire i morti per il vaccino. (university, center, Pos. 7520 – 7522)

Non avete ancora realizzato che se anche il Regime deciderà di ritirare il COVID PASS, di farvi tornare a lavorare, siete comunque già diventati cittadini di un Regime totalitario? Cittadini di uno schifosissimo Regime basato sulla menzogna, sulla pogressiva eliminazione delle libertà, sulla soppressione violenta del dissenso? (generic, Pos. 2127)

il "green pass non può essere richiesto poiché discriminatorio, lesivo della privacy e viola i seguenti articoli di Legge: - Art. 187 del Regolamento TULPS: un esercente commerciale è obbligato ad accogliere nel proprio esercizio qualsiasi persona, senza discriminazione pena ammenda fino a €3000,00.- Legge sulla Privacy: nessuno può obbligarci a fornire informazioni sulle nostre condizioni di salute.- Art. 120 Costituzione italiana: nessuno può limitare la libertà di movimento dell’individuo nel territorio della repubblica italiana. - Art. 13 Costituzione italiana: nessuno può limitare la libertà personale senza che ci sia una disposizione dell’Autorità Giudiziaria su fatti che riguardano il singolo individuo. (generic, Pos. 3448)

Aggiungi che rispetteremo tutte le misure di sicurezza anti covid ( distanziamento sociale ,igienizzazione,mascherina). Per quanto riguarda il riferimento a leggi e trattati,non vogliamo citare la convenzione dei diritti umani,il trattato di Oviedo e la sentenza della Cassazione che afferma che la salute del singolo non può essere sacrificata per la salute collettiva?non vogliamo accennare al principio di autodeterminazione? (university, center, Pos. 395 – 397)

Guarda al social score system cinese per capire la direzione folle di queste azioni, tipiche di sistemi dittatoriali e non di democrazie avanzate (university, south, Pos. 3755)

Oramai io credo che sta gente sia lobotomizzata e probabilmente la parola LIBERTÀ non sa manco cosa (university, south, Pos. 1255)

Se la risposta alla domanda è, che la vita è più importante della libertà allora tutte le leggi liberticide effettuate fino ad ora sono giustificabili e direi quasi giuste, arrivo a comprendere anche perché il green pass, legge palesemente discriminatoria, sia considerata giusta da molti.

Se la risposta alla domanda è la libertà è evidente che tutto ciò che è stato fatto fino ad ora viene considerato un errore a prescindere se una determinata legge sia stata fatta per salvare delle vite umane.

Arriviamo all’ultima risposta, quella almeno per me, più equilibrata, che la vita e la libertà hanno la stessa importanza. In vista di questa risposta è evidente che prendere delle precauzioni, per limitare il contagio ed i morti, è una cosa giusta e doverosa, quindi saranno inevitabili delle limitazioni (tipo distanziamento sociale, mascherine al chiuso, limitare i posti a sedere ecc.), ma a tempo stesso è importante preservare le libertà di tutti i cittadini. (university, center, Pos. 14996 – 15012)

Che razza di schifosissimo incubo vogliamo lasciare noi in eredità ai nostri figli? Un Regime sanitario? Un Regime che brutalizza ogni giorno le menti e i corpi dei suoi cittadini? Basta! Ribellatevi! (generic, Pos. 2127)

stiamo vivendo in una dittatura sanitaria e un autoritarismo politico che vanno comunque contrastati . Mi chiedo un generale a fare da commissario che se ne esce con parole assurde di voler stanare casa per casa i non 'vaccinati ' ? Questa gente se ne deve andare dal governo. Dobbiamo pretendere di andare di nuovo al voto. (university, center, Pos. 5904 – 5906)

### Action plan

È una questione politica ovunque. Se capiamo questo sappiamo chi dobbiamo combattere, è non è certo un virus. (university, north, Pos. 20112)

abbiamo potuto appurare l'intensa attività di dossieraggio anche di gruppi telegram. Insomma, adesso che le adesioni crescono, serve un minimo di 'arte della arte della guerra' (anzi della strategia, giusto per non dare il fianco alle accuse di terrorismo) (university, north, Pos. 20233)

niente disquisizioni che vadano oltre il tema da difendere come l'esistenza o meno del virus, la diatriba no-pro vax, il forum Davis, la depopolazione, la sperimentazione di massa, le varianti, i danni ecc. Sono tutti argomenti su cui si è bruciata l'autorevolezza di molti personaggi in vista, dato che rientrano facilmente nelle etichette per così dire 'disinnescanti' (complottista, laurea su Google, no Mask, no vax, no tamp, negazionista). (university, north, Pos. 3607)

Facciamo un flash mob in cui tutti i non vaccinati entrano tutti in contemporanea dove non possono? Magari correndo in modo da essere sudati (così hanno paura di toccarci) magari con in cappello che dice"l'ebreo si ribella" (generic, Pos. 1007)

firmerete davanti agli occhi increduli del vostro datore di lavoro la vostra dichiarazione di lotta non violenta. La vostra dichiarazione di Sciopero Generale ad Oltranza. Punto. Non serve altro. Saremo 100mila, e bloccheremo l'Italia, gli uffici, i servizi, la produzione. Staccheremo la spina a questo Regime infame. (generic, Pos. 2127)

### Summary: explaining green pass opposition without involving vaccines

La tesi principale deve continuare ad essere il fatto che si deve essere liberi di rifiutare un'iniezione, qualunque essa sia. Il corpo è mio e decido io. E se vi convincessero che il siero previene per l'x% il contagio (come alcuni provano ad insinuare) tutta la nostra battaglia cadrebbe? Credo proprio di no. (university, north, Pos. 24367)

### Other aspects: COVID-19

Inoltre rispondendo alla patetica provocazione vorrei sottolineare: il 99% dei decessi covid sono di ultraottantenni pluripatologici. (university, center, Pos. 2199 – 2202)

il COVID c‘é ma non si può fermare il mondo per questo. É una fottuta influenza, specialmente sui giovani. É morta molta più gente di influenza e non se ne é mai parlato (university, north, Pos. 2864)

Ti rendi conto che stai parlando di un virus di cui nessuno in nessuna parte del mondo riesce a dimostrare l’esistenza? (university, north, Pos. 1328)

il virus non é mai stato isolato né purificato. (university, north, Pos. 6509)

IL VIRUS È SOLO UN MEZZO PER IL RAGGIUNGIMENTO DI ALTRI OBBIETTIVI CHE NON CENTRANO NULLA CON LA TUTELA DELLA SALUTE (university, center, Pos. 8092 – 8095)

In più il più importante medico che abbiamo in Italia, dott. Remuzzi con H index 189, ha stilato da tempo un approvato protocollo di cura. Vada sul sito dell'istituto [name] Negri e si informi. C'è da aggiungere anche il dott. Scoglio candidato al Nobel 2018. (university, center, Pos. 14640 – 14643)

il covid si può curare a casa, con dei farmaci. C'è un gruppo di medici volontari che si occupano proprio di questo. Terapie domiciliari covid, è un gruppo fb molto seguito. (university, south, Pos. 1974)

Ascoltate anche dr Citro dr [name] Montanari dott.ssa Bolgan cosa dicono fanno fatto vaccinare le persone con la paura e con il ricatto sui giovani con il green pass. Ci molte reazioni avverse e non lo dicono resistete per il bene vostro. (university, center, Pos. 4198 – 4200)

La mascherina non protegge dai virus. Crea invece colonie di batteri che vi respirate oltre a porcherie che non vi dico per non passare x complottista. Giuste le osservazioni del collega sulla dott.ssa Gatti. Una grande nanopatologa. (university, north, Pos. 742)

Secondo il dott.Delgado non é un virus a provocare la malattia. Questo ve lo spiego quando ci incontriamo (university, north, Pos. 3485)

### Other aspects: Preferred measures

Esattamente, bisogna rispettare tutte le regole per impedire il contagio e quindi mascherine e distanziamento (university, south, Pos. 1467)

se vogliamo essere realmente certi che il virus non si diffonda in università, non dovrebbe essere utilizzato il tampone per chiunque entri in università, essendo l’unico strumento ad alta percentuale di riscontro della presenza del virus? (university, north, Pos. 25297)

Vorrei però che venisse garantita la didattica mista, sia in presenza che online, almeno nel primo semestre in modo da non aumentare il rischio di contagi e permettere a tutti di vaccinarsi. Per com'è la situazione a [place], con i trasporti e tutto quanto, è troppo alto il rischio di contagio anche per chi, da vaccinato, possa essere portatore. Non mi sento di prendermi la responsabilità di stare in giro a [place], anche eventualmente da vaccinato, e mettere a repentaglio la vita di altre persone. (university, center, Pos. 2095 – 2102)

Nonostante la fatica per raggiungere l'università, non è Università quella online, fatta di persone, sguardi, dialoghi CONCRETI; sono proprio la fatica e il tempo impiegati per andare all'università che sanciscono il suo valore fondante e formativo. La didattica a distanza non è un mezzo culturale adeguato. (university, north, Pos. 19204)

il tampone diventa uno strumento economicamente limitante per l’individuo, non essendo per nulla garantita agli studenti universitari la gratuità di questo servizio, con conseguente peso economico su chi sceglie di non vaccinarsi. (university, north, Pos. 25298)

### Other aspects: Anti-test and anti-mask positions

Io non ho ancora capito...(e' retorico e sarcastico) perche' per il virus piu' contagioso che si diffonde con una sola gocciolina macche' coll'aerosol, per aria...bisogna bucare fino alla barriera encefalica e fino alla ghiandola pineale? Forse perche' altrimenti non assimili l'ossido di grafene & chissa' cos'altro? In veterinaria si usa da tempo vaccinare per via nasale. Ps. Ci sono stati casi di rinoliquorrea ovvero perdita di liquido cerebrospinale, vertigini, emicranie anomali ecc, ovviamente come con il resto tutto viene puntualmente insabbiato e minimizzato (university, north, Pos. 11697-11698)

La mascherina non protegge dai virus. Crea invece colonie di batteri che vi respirate oltre a porcherie che non vi dico per non passare x complottista. Giuste le osservazioni del collega sulla dott.ssa Gatti. Una grande nanopatologa. (university, north, Pos. 742)

### Other aspects: Reliance on anecdotal evidence

Mio nonno è morto con il covid. Abbiamo seguito ciò che dicevano i medici delle cure a casa per mia nonna. Lei è sopravvissuta. Mio nonno ha voluto seguire la prassi invece. 2 settimane peggioramento. Terapia intensiva e morte. (university, center, Pos. 13863 – 13866)

Ho parlato con una dottoressa di [place]. Sapete cosa fanno per far credere che in terapia intensiva ci sono solo i non vaccinati? Quando arrivano pazienti covid, anche vaccinati con due dosi, hanno l’ordine di spostare i vaccinati in altri reparti e di lasciare i non vaccinati in terapia intensiva. (university, north, Pos. 24524)
