# Supplementary material for: Concerns Around Opposition to the Green Pass in Italy: Social Listening Analysis by Using a Mixed Methods Approach
Source: J Med Internet Res. 2022 Feb 16;24(2):e34385. doi: 10.2196/34385 (PMC8852653; doi:10.2196/34385)
Supplement: Multimedia Appendix 3 [file jmir_v24i2e34385_app3.docx]

Supplementary material: categories and topics included in the qualitative analysis

| **Category** | **Topic** | **Subcorpus** | **Pos** |
| --- | --- | --- | --- |
| Green pass and vaccines | ‘Stay on topic’ | University, south | 742 |
|  | ‘How can we ignore vaccines?’ | University, north | 6693 |
|  | ‘We cannot ignore vaccines’ | University, south | 1807 |
|  |  | University, center | 14716 – 14718 |
|  | Critical beliefs about vaccines | University, center | 3572 – 3579 |
|  | Fear of side effects | University, north | 25293 – 25294 |
|  |  | University, center | 15682 – 15688 |
|  | ‘Vaccines do not work’ | University, north | 2612 |
|  |  | University, north | 3385 |
|  | ‘Vaccines are part of a bigger scheme’ | University, south | 2343 |
|  |  | Generic | 72471 |
| Beyond vaccines: green pass, legal aspects and personal freedom | ‘The green pass is illegal and discriminatory’ | University, center | 3572 |
|  |  | University, center | 7520 – 7522 |
|  | ‘The green pass is part of a bigger scheme’ | Generic | 2127 |
|  | Jurisprudential arguments against the green pass | Generic | 3448 |
|  |  | University, center | 395 – 397 |
|  | Consequentialist arguments against the green pass | University, south | 3755 |
|  |  | University, south | 1255 |
|  | Deontological arguments against the green pass | University, center | 14996 – 15012 |
|  | Converging no-green-pass arguments | Generic | 2127 |
|  |  | University, center | 5904 – 5906 |
| Action plan | Who are the real enemies? | University, north | 20112 |
|  | ‘Avoid defusing topics’ | University, north | 20233 |
|  |  | University, north | 3607 |
|  | Protests | Generic | 1007 |
|  |  | Generic | 2127 |
| Explaining green pass opposition without involving vaccines | ‘The body is mine and I decide’ | University, north | 24367 |
| What is COVID-19? | ‘COVID-19 is just a flu’ | University, center | 2199 - 2202 |
|  |  | University, north | 2864 |
|  | ‘COVID-19 does not exist’ | University, north | 1328 |
|  |  | University, north | 6509 |
|  | ‘COVID-19 is a means to other ends’ | University, center | 8092 – 8095 |
| Expertise | ‘Our experts’ | University, center | 14640 – 14643 |
|  |  | University, south | 1974 |
|  |  | University, center | 4198 – 4200 |
|  |  | University, north | 742 |
|  |  | University, north | 3485 |
| Preferred measures | Masks, distancing, tests, dual teaching | University, south | 1467 |
|  |  | University, north | 25297 |
|  |  | University, center | 2095 – 2102 |
|  | Against online teaching and tests | University, north | 19204 |
|  |  | University, north | 25298 |
| Anti – test and anti – mask positions | ‘Testing is dangerous’ | University, north | 11697 – 11698 |
|  | ‘Masks do not work’ | University, north | 742 |
| Reliance on anecdotal evidence | ‘If it’s personal it’s true’ | University, center | 13863 – 13866 |
|  |  | University, north | 24524 |
