# Supplementary material for: Concerns Around Opposition to the Green Pass in Italy: Social Listening Analysis by Using a Mixed Methods Approach
Source: J Med Internet Res. 2022 Feb 16;24(2):e34385. doi: 10.2196/34385 (PMC8852653; doi:10.2196/34385)
Supplement: Multimedia Appendix 4 [file jmir_v24i2e34385_app4.docx]

#### Multimedia appendix 4

| Number | Lemma, symbol or expression | Frequency (%) |
| --- | --- | --- |
| 1 | 😂 | 18.1 |
| 2 | Know (sapere) | 5.9 |
| 3 | Want (volere) | 5.5 |
| 4 | Can (potere) | 4.7 |
| 5 | 🤣 | 4.3 |
| 6 | Must (dovere) | 4.1 |
| 7 | Ok | 3.4 |
| 8 | Take (prendere) | 3.4 |
| 9 | Hello (ciao) | 3.2 |
| 10 | See (vedere) | 3.1 |
| 11 | Put (mettere) | 3.1 |
| 12 | Do (fare) | 2.6 |
| 13 | Say (dire) | 2.6 |
| 14 | Ah | 2.5 |
| 15 | 🌚 | 2.5 |
| 16 | 2 | 2.5 |
| 17 | Parrot (pappagallo) | 2.4 |
| 18 | ❤ | 2.4 |
| 19 | Cage (gabbia) | 2.4 |
| 20 | Go (andare) | 2.3 |

The 20 most used lemmas, symbols, or expressions in control chats. The table lists the most frequently used lemmas), symbols or expressions (in percentage) on average across each individual control chat (n=5).
