# Supplementary material for: Concerns Around Opposition to the Green Pass in Italy: Social Listening Analysis by Using a Mixed Methods Approach
Source: J Med Internet Res. 2022 Feb 16;24(2):e34385. doi: 10.2196/34385 (PMC8852653; doi:10.2196/34385)
Supplement: Multimedia Appendix 5 [file jmir_v24i2e34385_app5.docx]

#### Multimedia appendix 5

| Number | Lemma, symbol or expression | Frequency (%) |
| --- | --- | --- |
| 1 | Can (potere) | 9.3 |
| 2 | Green | 9.2 |
| 3 | Link | 8.6 |
| 4 | Pass | 7.5 |
| 5 | Must (dovere) | 5.5 |
| 6 | Want (volere) | 5.3 |
| 7 | Know (sapere) | 4.5 |
| 8 | Freedom (libertà) | 4.1 |
| 9 | Ask (chiedere) | 4.0 |
| 10 | Vaccine (vaccino) | 3.8 |
| 11 | Message (messaggio) | 3.8 |
| 12 | Do (fare) | 3.5 |
| 13 | Law (legge) | 3.5 |
| 14 | Article (articolo) | 3.4 |
| 15 | Hashtag | 3.1 |
| 16 | Shop owner (esercente) | 2.8 |
| 17 | Say (dire) | 2.8 |
| 18 | Speak (parlare) | 2.6 |
| 19 | Square (piazza) | 2.5 |
| 20 | Come (venire) | 2.5 |
