# Supplementary figures and images for: Concerns Around Opposition to the Green Pass in Italy: Social Listening Analysis by Using a Mixed Methods Approach
Source: J Med Internet Res. 2022 Feb 16;24(2):e34385. doi: 10.2196/34385 (PMC8852653; doi:10.2196/34385)

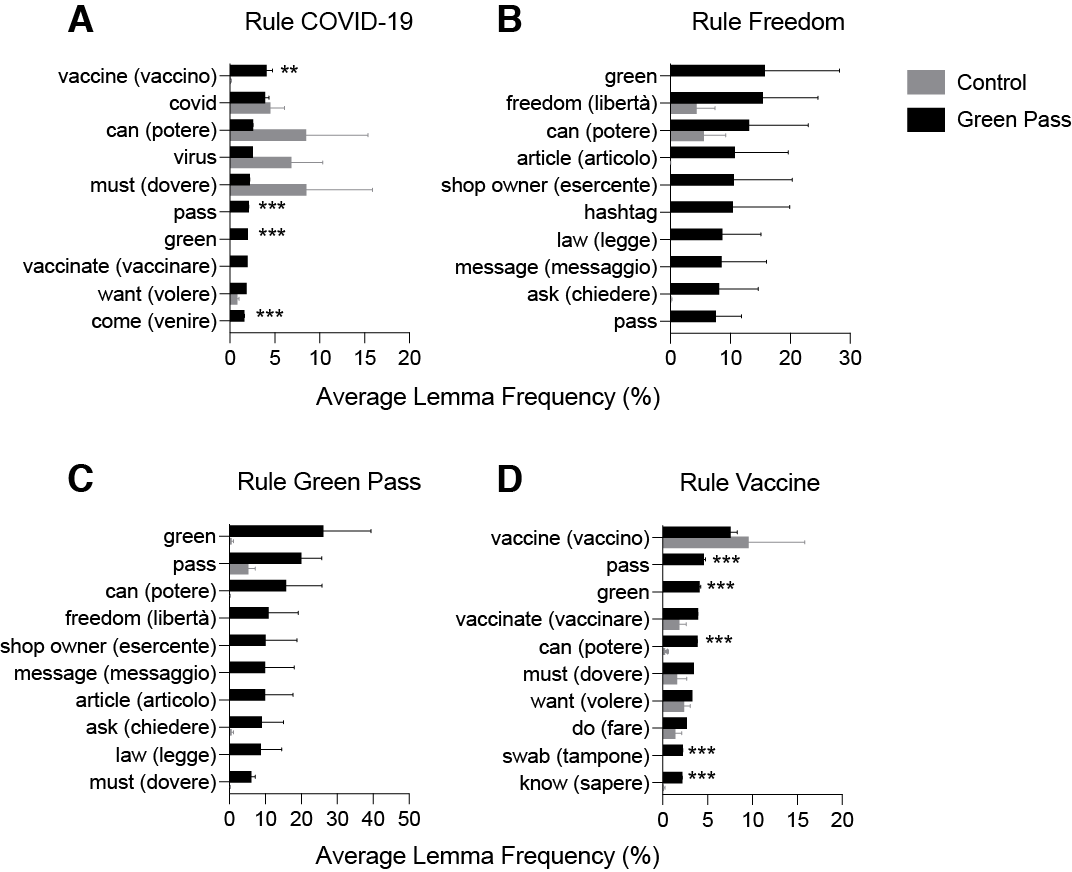

Supplement: Multimedia Appendix 6 [file jmir_v24i2e34385_app6.png]
